# Insights into the Host Range, Genetic Diversity, and Geographical Distribution of Jingmenviruses

**DOI:** 10.1128/mSphere.00645-19

**Published:** 2019-11-06

**Authors:** Sarah Temmam, Thomas Bigot, Delphine Chrétien, Mathilde Gondard, Philippe Pérot, Virginie Pommelet, Evelyne Dufour, Stéphane Petres, Elodie Devillers, Thavry Hoem, Valérie Pinarello, Vibol Hul, Khamsing Vongphayloth, Jeffrey C. Hertz, Irène Loiseau, Marine Dumarest, Veasna Duong, Muriel Vayssier-Taussat, Marc Grandadam, Emmanuel Albina, Philippe Dussart, Sara Moutailler, Julien Cappelle, Paul T. Brey, Marc Eloit

**Affiliations:** a Institut Pasteur, Biology of Infection Unit, Inserm U1117, Pathogen Discovery Laboratory, Institut Pasteur, Paris, France; b Institut Pasteur—Bioinformatics and Biostatistics Hub—Computational Biology Department, USR 3756 CNRS, Institut Pasteur, Paris, France; c UMR ASTRE, CIRAD, INRA, Université de Montpellier, Montpellier, France; d CIRAD, UMR ASTRE, Petit-Bourg, Guadeloupe, France; e UMR BIPAR, Animal Health Laboratory, ANSES, INRA, Ecole Nationale Vétérinaire d’Alfort, Université Paris-Est, Maisons-Alfort, France; f Institut Pasteur du Laos, Vientiane, Lao People's Democratic Republic; g Institut Pasteur, Production and Purification of Recombinant Proteins Technological Platform—C2RT, Institut Pasteur, Paris, France; h Virology Unit, Institut Pasteur du Cambodge, Institut Pasteur International Network, Phnom Penh, Cambodia; i U.S. NAMRU 2, NMRCA, Singapore; j Epidemiology and Public Health Unit, Institut Pasteur du Cambodge, Institut Pasteur International Network, Phnom Penh, Cambodia; k UMR EpiA, INRA, VetAgro Sup, Marcy l'Etoile, France; l National Veterinary School of Alfort, Paris-Est University, Maisons-Alfort, France; University of Texas Southwestern Medical Center

**Keywords:** Jingmenvirus, LIPS, emergence, evolution

## Abstract

Jingmenvirus is a recently identified group of segmented RNA viruses phylogenetically linked with unsegmented *Flaviviridae* viruses. Primarily identified in various tick genera originating in China, Jingmenvirus geographical distribution has rapidly expanded to cover Africa, South America, Caribbean, and Europe. The identification of Jingmen-related viruses in various mammals, including febrile humans, opens the possibility that Jingmenviruses may be novel tick-borne arboviruses. In this study, we aimed at increasing knowledge of the host range, genetic diversity, and geographical distribution of Jingmenviruses by reporting for the first time the identification of Jingmenviruses associated with Rhipicephalus microplus ticks originating in the French Antilles (Guadeloupe and Martinique islands), with Amblyomma testudinarium ticks in Lao PDR, and with Ixodes ricinus ticks in metropolitan France, and from urine of Pteropus lylei bats in Cambodia. Analyses of the relationships between the different Jingmenvirus genomes resulted in the identification of three main phylogenic subclades, each of them containing both tick-borne and mammal-borne strains, reinforcing the idea that Jingmenviruses may be considered as tick-borne arboviruses. Finally, we estimated the prevalence of Jingmenvirus-like infection using luciferase immunoprecipitation assay screening (LIPS) of asymptomatic humans and cattle highly exposed to tick bites. Among 70 French human, 153 Laotian human, and 200 Caribbean cattle sera tested, only one French human serum was found (slightly) positive, suggesting that the prevalence of Jingmenvirus human and cattle infections in these areas is probably low.

**IMPORTANCE** Several arboviruses emerging as new pathogens for humans and domestic animals have recently raised public health concern and increased interest in the study of their host range and in detection of spillover events. Recently, a new group of segmented *Flaviviridae*-related viruses, the Jingmenviruses, has been identified worldwide in many invertebrate and vertebrate hosts, pointing out the issue of whether they belong to the arbovirus group. The study presented here combined whole-genome sequencing of three tick-borne Jingmenviruses and one bat-borne Jingmenvirus with comprehensive phylogenetic analyses and high-throughput serological screening of human and cattle populations exposed to these viruses to contribute to the knowledge of Jingmenvirus host range, geographical distribution, and mammalian exposure.

## Abstract (precis)

Several arboviruses emerging as new pathogens for humans and domestic animals have recently raised public health concern and increased interest in the study of their host range and in detection of spillover events. Recently, a new group of segmented *Flaviviridae*-related viruses, the Jingmenviruses, has been identified worldwide in many invertebrate and vertebrate hosts, pointing out the issue of whether they belong to the arbovirus group. The study presented here combined whole-genome sequencing of three tick-borne Jingmenviruses and one bat-borne Jingmenvirus with comprehensive phylogenetic analyses and high-throughput serological screening of human and cattle populations exposed to these viruses to contribute to the knowledge of Jingmenvirus host range, geographical distribution, and mammalian exposure.

## INTRODUCTION

Jingmenviruses are a recently reported group of enveloped, positive-sense ssRNA viruses as yet unassigned to a viral family or genus (1). Their genome is composed of four segments: two segments coding for nonstructural (NS) proteins and presenting homologies with flavivirus nonstructural proteins 3 (NS3) and 5 (NS5), while structural proteins have no known homologs (2) and are thought to have originated from an as-yet-undiscovered ancestral virus (3). The prototype strain, Jingmen tick virus (JMTV) strain SY84, was previously reported to be primarily associated with cattle-infesting Rhipicephalus microplus ticks in China (2). However, knowledge of the geographical distribution and host range of JMTV-like viruses has rapidly expanded with the identification of closely related viruses in R. microplus ticks originating from China (2), Brazil (4), and Trinidad and Tobago (5); in Chinese *Haemaphysalis* sp., *Ixodes* sp., Dermacentor nuttalli (Yanggou tick virus), and Amblyomma javanense ticks (2, 3); in *Anopheles*, *Aedes*, *Culex*, and *Armigeres* mosquitoes originating from China (2, 6); in Ixodes ricinus ticks originating from Finland (7); in R. geigyi ticks (Kindia tick virus) originating from Guinea; in Ugandan primates (8); and in Chinese and Brazilian cattle (2, 9). Maruyama et al. and, more recently, Jia et al. (3, 4) reported the identification of JMTV in salivary glands of R. microplus ticks, highlighting their probable role as vectors in JMTV transmission to vertebrates. More distantly related viruses presenting similar characteristics with respect to genome organization and phylogenetic relatedness to JMTV in samples from various hematophagous and nonhematophagous insects (fleas, mosquitoes, crickets, aphids, etc.) were also reported previously (1, 8). In humans, viruses closely related to JMTV were found to be primarily associated with patients in Kosovo presenting with Crimean-Congo hemorrhagic fever infection, reflecting their exposure to tick bites (10), but without any information on JMTV pathogenicity. More recently, two studies simultaneously reported the identification of Jingmen-related viruses in Chinese patients with a history of tick bites manifesting in unexplained febrile illness (3, 6), suggesting that JMTV might be responsible for those symptoms and hence might represent a novel tick-borne human pathogen.

In this study, we aimed at increasing the knowledge of the host range and geographical distribution of Jingmenviruses (i) by reporting the identification and full-genome sequencing of JMTV-like viruses associated with Rhipicephalus microplus ticks originating from the French Antilles (Guadeloupe and Martinique French overseas territories), with Amblyomma testudinarium ticks from Lao People's Democratic Republic (Lao PDR), and with Ixodes ricinus ticks from metropolitan France, as well as in urine of Pteropus lylei bats from Cambodia and (ii) by using luciferase immunoprecipitation system (LIPS)-based serological screening of humans and cattle exposed to tick bites in France, Guadeloupe, and Lao PDR to determine the prevalence of JMTV-like infection in asymptomatic humans and cattle.

## RESULTS

### Increasing host range and geographical distribution of Jingmenviruses.

**FIG 1:**
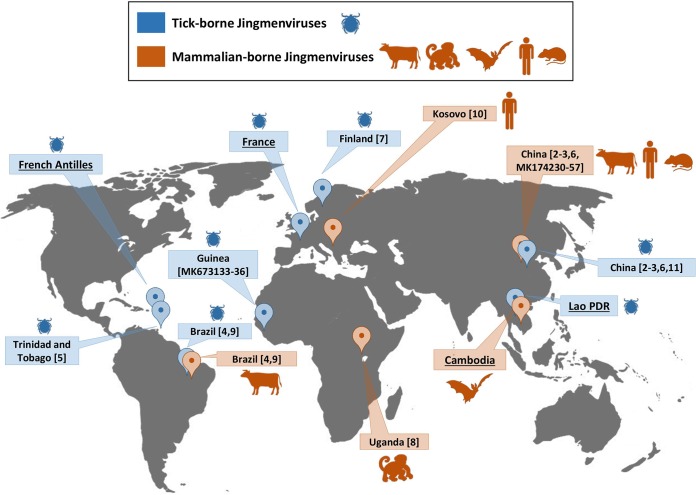
Reports of arbo-Jingmenviruses according to invertebrate (blue) or vertebrate (orange) host. The names of the countries of origin of the viruses described in the present study are underlined. Numbers in square brackets refer to referenced articles or virus segment or both.

**FIG 2:**
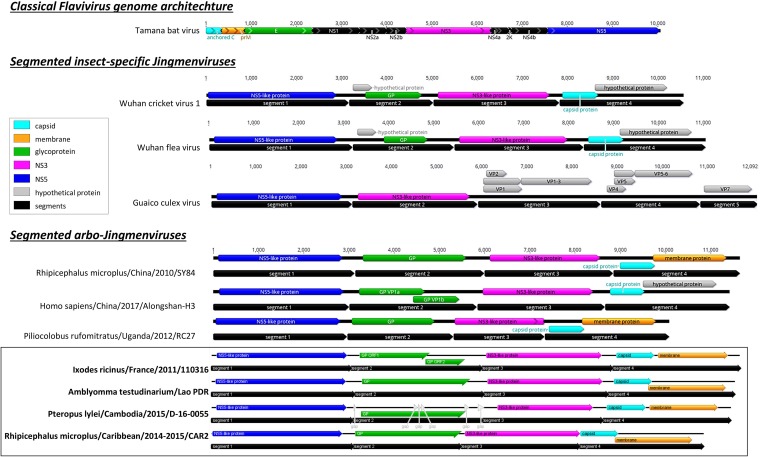
Genome organization of representative insect-specific and arbo-Jingmenviruses and genome organization of JMTV determined in this study. Segments (in black) were concatenated for better clarity. Light blue, capsid genes; orange, membrane; green, glycoprotein; pink, NS3; dark blue, NS5; gray, hypothetical proteins. The sequences in the box are those determined in this study. Gaps in Cambodian bat-borne JMTV are highlighted by white arrowheads.

Jingmen tick virus (JMTV) *sensu stricto* was first identified in various arthropods (including in *Rhipicephalus* sp., *Haemaphysalis* sp., A. javanense, *Ixodes* sp., and D. nuttalli ticks in China, Brazil, Trinidad and Tobago, Guinea, and Finland [2–7, 9, 11] and in various mosquito species in China [2, 6]). In mammals, JMTV was identified in humans in Kosovo and China, in cattle in Brazil, and in primates in Uganda (2, 3, 6, 8–10) (Fig. 1). We report here the detection of JMTV-related sequences in I. ricinus, R. microplus, and A. testudinarium ticks originating from metropolitan France, French Antilles, and Laos, respectively, and in a pool of urine specimens from frugivorous Pteropus lylei bats originating from Cambodia. Table 1 presents some metrics of the next-generation sequencing (NGS) data sets from which JMTV-like sequences were identified. Except for bat-borne JMTV, for which internal small gaps were found in segment 2 glycoproteins (GP) due to issues in finishing the viral genome (Fig. 2), the complete open reading frames (ORFs) of the four segments of JMTV were obtained for the four JMTV strains, with mean coverage per base ranging from 3.44× (bat-borne JMTV) to 7,550× (French tick-borne JMTV). The presence of Jingmenvirus-related viral RNA in each original sample was confirmed by quantitative reverse transcription-PCR (RT-qPCR) amplification targeting the polymerase (segment 1) gene followed by Sanger sequencing (see Fig. S1 in the supplemental material). To verify that the identified JMTV sequences did not correspond to endogenous viral sequences integrated in the genome of ticks and bats, the same qPCR targeting the polymerase gene was performed in a nested format without RT and gave negative results for each Jingmen strain (Fig. S1). Of note, the JMTV genome from French Antilles ticks is a consensus of genomes originating in a pool of R. microplus and A. variegatum ticks. Individual RT-PCR screenings of ticks revealed that both species were infected, with a higher prevalence in R. microplus than in A. variegatum ticks (42% versus 5%) (M. Gondard, S. Temmam, E. Devillers, V. Pinarello, T. Bigot, D. Chrétien, R. Aprelon, M. Vayssier-Taussat, E. Albina, M. Eloit, and S. Moutailler, unpublished data), which probably reflects cofeeding of these two tick genera on the same cattle host, as previously suggested (12). Therefore, the French Antilles JMTV strain is referred here as a (more likely) strain of R. microplus. Surprisingly, and although JMTV-related sequences were detected in French I. ricinus ticks from Alsace, no JMTV was identified in French I. ricinus ticks from Ardennes, an area located only 200 km from the Alsace sampling area. Similarly, JMTV was not detected in *Haemaphysalis* sp. ticks originating from Lao PDR, although JMTV-positive *Amblyomma* ticks were collected concomitantly at the same location (data not shown).

**TABLE 1:** Some metrics regarding the NGS transcriptome analyses

Sample source	No. of raw reads (paired)	No. of cleaned reads (paired)	No. of contigs	No. of singletons	Avg contig length (nt)	No. of viral sequences	No. of JMTV- like sequences
Ticks							
Ixodes ricinus, Alsace, France^*a*^	150,756,775	148,229,568	163,565	3,926,877	165	15,227	4,619
Rhipicephalus microplus, French Antilles	41,696,475	41,581,009	28,565	1,188,734	168	700,252	258,023
*Amblyomma* test*udinarium*, Lao PDR	53,106,358	53,101,135	28,749	3,103,834	162	3,801	3,548

Bats							
Urine 1, *Pteropus lylei*, Cambodia	69,925,441	69,760,609	112,609	5,703,210	144	607	0
Urine 2, *Pteropus lylei*, Cambodia	66,479,314	66,476,147	309,632	1,075,945	143	111,836	754

a Two independent sequencing runs were performed for the Alsace sample; metrics correspond to the 2 runs.

10.1128/mSphere.00645-19.1 FIG S1 Agarose gel electrophoresis of PCR targeting putative JMTV endogenous viral elements. “DNA” refers to total nucleic acids without an RT step; “RNA” refers to total nucleic acids with an RT step. Arrows highlight expected size bands. Download FIG S1, TIF file, 0.1 MB. Copyright © 2019 Temmam et al. 2019 Temmam et al. This content is distributed under the terms of the Creative Commons Attribution 4.0 International license.

### Genetic diversity of Jingmenviruses.

**FIG 3:**
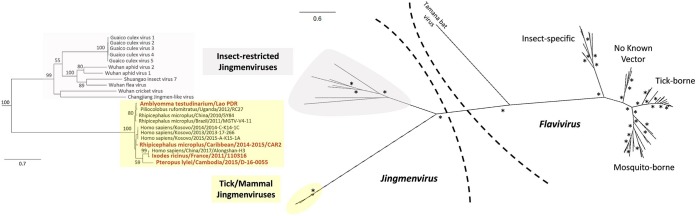
ML phylogenetic reconstruction of the complete NS5 amino acid sequences of Jingmenviruses and representative *Flavivirus* genomes. The names of the viruses described in the present study are indicated in bold. A bootstrap value above 90 is highlighted by an asterisk (*).

Tick-borne Jingmenvirus genome organization presents three main characteristics: (i) the segmentation; (ii) the monocistronic or bicistronic expression patterns; and (iii) the presence of two key nonstructural viral proteins that are shared with *Flaviviridae*. Similar genomic organization and expression patterns were reported for phylogenetically distant Jingmenviruses described in fleas, mosquitoes, crickets, and aphids by Ladner (8) and Shi (1) (Fig. 2). We performed a phylogenetic reconstruction of the complete RNA-dependent RNA polymerase (RdRP) of these viruses in addition to other Jingmenviruses and representative *Flavivirus* genomes, resulting in classification of Jingmenviruses into two distinct clades: one is an insect-restricted clade, while the other is likely an arbovirus clade (Fig. 3). The latter clade comprises tick-borne JMTV identified in samples either from various tick vectors or from mammals, namely, primate, bat, and human. Interestingly, this arbovirus Jingmenvirus clade seems to divide into different subclades, among which the French I. ricinus, the Cambodian *Pteropus*, and the Chinese human JMTV isolates to form a distinct group of viruses.

**FIG 4:**
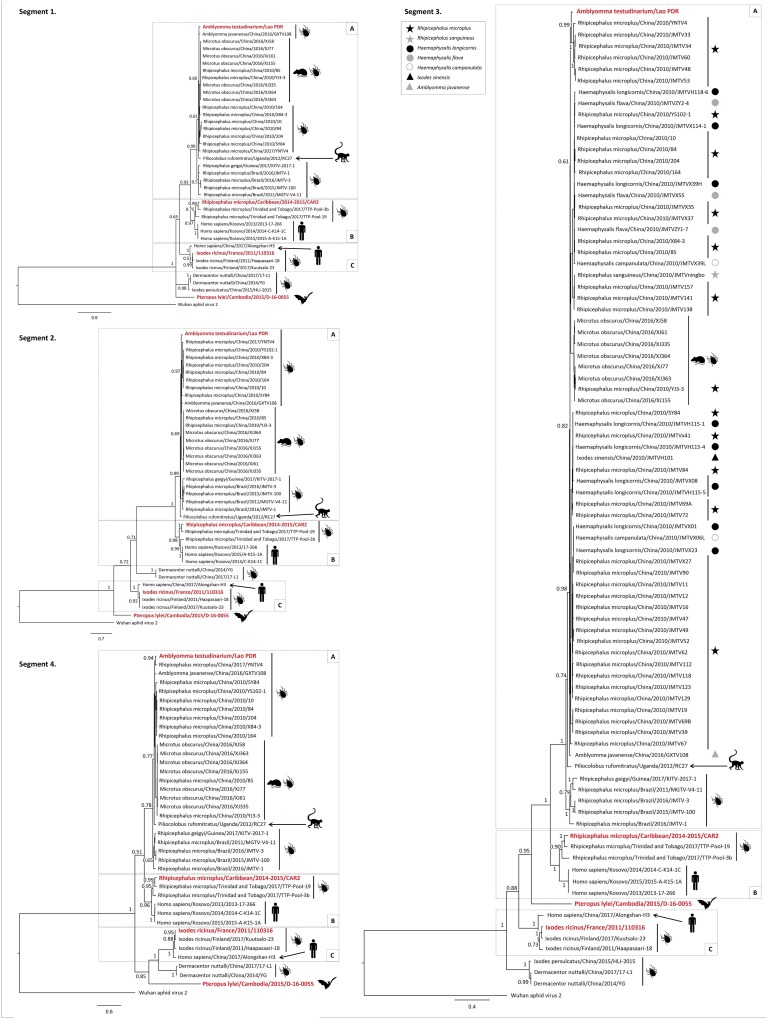
Bayesian phylogenetic reconstruction of the four nucleotide segments of arbo-Jingmenviruses according to host. For segment 3, Chinese *Rhipicephalus* ticks are represented by a star (black for R. microplus, gray for R. sanguineus), *Haemaphysalis* by a circle (black for H. longicornis, gray for H. flava, white for H. campanulata), and *Ixodes sinensis* by a black triangle. The viruses described in the present study are highlighted in red. Accession numbers of sequences used in this analysis are provided in Table S1. Posterior probabilities above 0.5 are indicated.

To confirm this observation and to identify putative events of reassortment between JMTV strains that could have occurred during virus evolution, phylogenetic reconstructions were performed for each segment of the arbo-Jingmenviruses (Fig. 4; see also Table S1 and Fig. S2 in the supplemental material). The tree topologies were globally congruent for the different segments, except for Cambodian bat-borne Jingmenvirus. A minimum of three major clades are observed within the arbo-Jingmenviruses. Each clade contains both tick-borne and mammal-borne viruses. Clade A is composed of JMTV strains mainly isolated from Rhipicephalus microplus (originating from China, Guinea, and Brazil, with the Brazilian strains forming a distinct subclade) for the tick part and of JMTV strains isolated from Microtus obscurus Chinese rodents and Ugandan primate for the mammalian counterpart. The Laotian A. testudinarium strain of JMTV belongs to clade A, as does a Chinese A. javanense JMTV isolate. Interestingly, two sequences of Chinese tick-borne JMTV are embedded in the subclade of rodent-borne JMTV, suggesting frequent events of transmission between ticks and rodents and a possible role of reservoirs for rodents. Human JMTV-related viruses originating from Kosovo and tick-borne JMTV originating from French Antilles and from Trinidad and Tobago belong to clade B. Finally, clade C is composed of JMTV strains from I. ricinus originating from metropolitan France and Finland for the invertebrate part and of Chinese human Alongshan Jingmenvirus for the mammalian counterpart. Surprisingly, the Cambodian bat-associated strain is placed at different positions of the tree depending on the segment. For example, it is located at the root of clades A and B in segments 1 and 3 (in a trifurcation with clade C and D. nuttalli JMTV in segment 1 and independently from clade C in segment 3), whereas it is located at the root of all clades in segment 2. Similarly, bat-borne JMTV is located at the root of clade C in segment 4 (Fig. 4). These results suggest differences in the evolution rates of bat-borne Jingmenvirus segments.

10.1128/mSphere.00645-19.3 TABLE S1 Accession numbers of Jingmen-like nucleotide sequences according to host, location, and year of collection used in phylogenetic analyses. *, partial sequences. Blue, complete ORFs. Download Table S1, DOCX file, 0.02 MB. Copyright © 2019 Temmam et al. 2019 Temmam et al. This content is distributed under the terms of the Creative Commons Attribution 4.0 International license.

Interestingly, no clear clustering according to Chinese tick species was observed (segment 3, Fig. 4), indicating that frequent transmissions of JMTV strains occur between different tick species, possibly via cofeeding on a same mammalian host, as suggested by Shi et al. (1). Similarly, no clear clustering by geographical origin was observed for tick strains of Jingmenviruses. Despite the fact that clade B seems to contain tick-borne JMTV originating only in Caribbean (French Antilles and Trinidad and Tobago) and clade C tick-borne strains only in Europe (France and Finland), clade A contains tick-borne strains originating from Asia, Brazil, and Guinea, suggesting that dispersal over long distances was frequent during JMTV evolution, as previously reported (1).

Tick-borne Jingmenvirus genome organization is well conserved between strains belonging to the three subclades (Fig. 2; see also Table 2). Segment 1 codes for a unique ORF of 914 amino acids (aa) presenting homologies with *Flavivirus* NS5 (as shown in Fig. 3), which is in the range of those observed within the *Flavivirus* genus (897 to 905 aa). As for its flavivirus counterpart, NS5-like genes of Jingmenviruses code for the RNA-dependent RNA polymerase (RdRP) and the methyltransferase, as previously described (6). Segment 3 codes for a unique ORF corresponding to the second nonstructural (NS) protein of the virus (666 to 810 aa), which shares homology with the flavivirus NS3 protein. One should note that the structure of segments coding NS proteins of Jingmenviruses is remarkably well conserved among all subclades of Jingmenviruses (Table 2). Segments coding for the three structural proteins (the viral glycoprotein [GP] and capsid and membrane proteins), however, present more diversity in their genetic organization. For example, segment 2 coding for the viral GP presents a monocistronic or bicistronic expression pattern, depending on the phylogenetic clade to which the strains belong (e.g., viruses falling into clade C might present two ORFs coding for the GP whereas all isolates of clades A and B present a monocistronic GP). Similarly, segment 4 coding for the viral capsid and membrane proteins is bicistronic for strains belonging to the three subclades, but these two ORFs may overlap in some strains (Table 2). Interestingly, viruses belonging to subclades A and B seem to present more highly conserved genome organization (and protein length) between isolates than viruses belonging to clade C.

**TABLE 2:** Features of Jingmenviruses genome organization and expression strategy^*a*^

Characteristic	Result								
	Subclade A				Subclade B		Subclade C		Outgroup
	JMTV/ A. testudinarium/ Lao PDR	JMTV/ R. microplus/ China	JMTV/ primate/ Uganda	MGTV/ R. microplus/ Brazil	JMTV/ R. microplus/ French Antilles	JMTV/ human/ Kosovo	JMTV/ I. ricinus/ France	ALSV /human/ China	JMTV/ P. lylei/ Cambodia
Segment 1									
Accession no.	MN095519	KJ001579	KX377513	JX390986	MN095523	MH133313	MN095527	MH158415	MN095531
Length (nt)	3,070	3,114	2,950	2,963	3,044	2,962	2,992	2,994	3,025
No. of ORFs	1	1	1	1	1	1	1	1	1
RdRP length (aa)	914	914	914	914	914	914	914	914	914

Segment 2									
Accession no.	MN095520	KJ001580	KX377514	KY523073	MN095524	MH133315	MN095528	MH158416	MN095532*
Length (nt)	2,774	2,847	2,326	2,629	2,309	2,657	2,803	2,806	2,788
No. of ORFs	1	1	1	1	1	1	2	2	1
Overlapping ORFs?	NR	NR	NR	NR	NR	NR	Yes	Yes	NR
GP1 length (aa)	754	754	604	753	744	744	481	481	735
GP2 length (aa)	NR	NR	NR	NR	NR	NR	266	335	NR

Segment 3									
Accession no.	MN095521	KJ001581	KX377515*	JX390985	MN095525	MH133314	MN095529	MH158417	MN095533
Length (nt)	2,660	2,824	1,996	2,705	2,537	2,647	2,807	2,811	2,582
No. of ORFs	1	1	1	1	1	1	1	1	1
NS3 protein length (aa)	808	808	657	808	808	808	810	810	666

Segment 4									
Accession no.	MN095522	KJ001582	KX377516	KY523074	MN095526	MH133316	MN095530	MH158418	MN095534
Length (nt)	2,710	2,794	2,741	2,728	2,654	2,611	2,735	2,738	2,733
No. of ORFs	2	2	2	2	2	2	2	2	2
Overlapping ORFs?	Yes	Yes	Yes	no	Yes	Yes	no	Yes	No
Capsid length (aa)	254	254	254	254	254	254	252	252	265
Membrane length (aa)	538	538	538	502	538	538	484	538	471

a Characteristics of the newly described Jingmen genomes are mentioned along with several representative JMTV sequences. MGTV, Mogiana tick virus; ALSV, Alongshan virus; RdRP, putative RNA-dependent RNA polymerase; GP, putative glycoprotein; NR, not reported. An asterisk (*) indicates a partial sequence.

### Exposure to Jingmenviruses in two tick/mammal interfaces.

**FIG 5:**
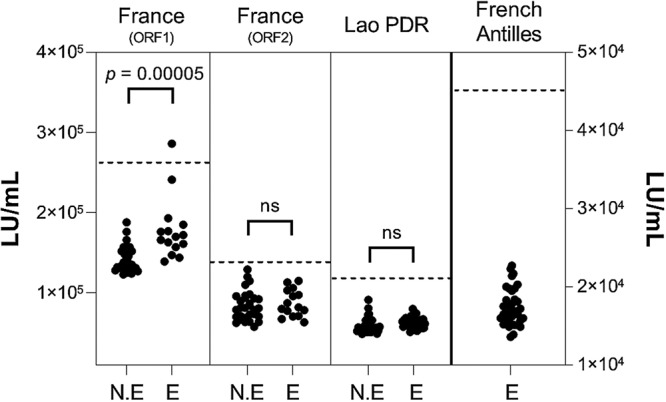
Distribution of luciferase activity data (indicated in light units per milliliter) after LIPS performed in tick/human and tick/cattle interfaces. NE, nonexposed human population; E, exposed human or cattle populations. A horizontal dashed line indicates the positivity threshold for each antigen construct. *t* test statistical analysis (α = 0.05) was used to compare the means of the number of light units per milliliter determined for the exposed and nonexposed human groups. ns, not statistically significant.

There is mounting evidence that Jingmenviruses constitute novel tick-borne arboviruses. We therefore analyzed two tick/human interfaces and one tick/cattle interface for the presence of specific JMTV antibodies in mammals by the use of a luciferase immunoprecipitation system (LIPS). Results are presented in Fig. 5.

Transmission of JMTV by Ixodes ricinus ticks to French human populations exposed to tick bites was evaluated with LIPS assay targeting the two predicted external domains of JMTV glycoprotein (GP). None of the serum samples exceeded the positivity threshold when the second domain (ORF2) was used as the antigen. One slightly positive French human serum sample and a significant difference in light unit (LU) means (*P = *0.00005) were observed between the two groups when GP ORF1 was used as the antigen (Fig. 5). Indeed, the mean luciferase activity was impacted by one serum sample measured at 2.86E + 05 LU/ml (when the positivity threshold was defined at 2.62E + 05 LU/ml). This serum sample belonged to the exposed group, suggesting that this person may have been exposed to JMTV. However, as we cannot exclude the possibility that this low level of positivity might have been due to the presence of cross-reactive antibodies, more-specific serological tests, such as seroneutralization, are needed to confirm this result, which would require an isolate of the virus.

The tick/human interface in Lao PDR mediated by A. testudinarium ticks as putative vectors did not reveal any serological trace of JMTV infection, either in exposed or in nonexposed human populations (Fig. 5). Similarly, none of the Guadeloupian cattle serum samples representing the French Antilles tick/cattle interface were above the positivity threshold (Fig. 5, right axis), although the prevalence of JMTV in Guadeloupian R. microplus ticks was found to be 30% to 40% among ticks collected on animals (Gondard et al., unpublished), suggesting that the conditions for sustained tick-borne JMTV transmission to cattle are not present in Guadeloupe.

## DISCUSSION

Emerging infectious diseases are described as infections that are newly appearing in a population or that have existed but are rapidly increasing in incidence or geographic range (13). Among them, emerging viruses could appear into human populations via two main routes of transmission: (i) increasing contacts between wildlife and human populations, leading to the spillover of zoonotic viruses, and (ii) geographical expansion of infected hematophagous arthropods or their vertebrate hosts that disseminate arboviruses from areas of endemicity to novel ecosystems, comprising vertebrates immunologically naive to these viruses and adequate vectors. The second route of transmission may expose large populations to new pathogens, as shown for mosquito-borne viruses (illustrated by Zika virus expansion into the Americas [14]), but is less likely for tick-borne arboviruses. However, fatal infections caused by severe fever with thrombocytopenia syndrome (SFTS) virus (15) and the recent reports of Jingmenviruses in febrile patients with unknown etiology (3, 6) illustrate well how novel (and previously unknown) tick-borne viruses may emerge in naive human populations.

Jingmenviruses represent the only group of *Flaviviridae*-related viruses with segmented genomes. The origin of this segmentation has been extensively described elsewhere (1, 2, 8). Both insect-restricted and arbo-Jingmenviruses are composed of four genomic segments, except for Guaico Culex virus, which is a 5-segmented multipartite mosquito-borne virus (with one segment being not essential for viral replication), which forms a distinct subclade within the insect-specific Jingmenviruses (Fig. 3) (8). The tick-borne French, French Antilles, and Laotian JMTV and bat-borne Cambodian Jingmenviruses described in this study follow this rule. Although segmented viruses are subject to reassortment events that contribute to the macroevolution of these viruses (16, 17), the Jingmenvirus genome is extremely stable among vertebrate and invertebrate hosts, suggesting that the virus is already well adapted to both hosts (Table 2). Indeed, we did not observe any obvious mutation that could reflect the adaptation of the virus to a vertebrate or invertebrate host. Another example of evidence of this low level of macroevolution is their remarkable genome conservation among geographically distant strains, as demonstrated by Jingmenviruses strains belonging to clade A that originated from Asia, Africa, and South America (Fig. 4). Together, these observations suggest that the high level of stability of Jingmen arboviruses is more likely due to dispersals of the same virus over long distance. The role of migratory birds (18), rodents (19), or domestic animals (20) that could be infested by ticks or viremic or both has to be investigated to better understand the dissemination of Jingmenviruses over continents. Similarly, the role of bats in the environmental cycle of Jingmenviruses has to be evaluated. Indeed, Pteropus lylei bats were shown to be able to switch among roosts separated by up to 105 km, although they are not subject to seasonal migrations, in contrast to other *Pteropus* bats (21, 22), indicating that bats might also contribute to local and regional spread of the virus (23).

At a more local scale, the ecological cycle of Jingmenviruses seems to be permissive to different arthropod hosts. Indeed, the wide range of distribution of arbo-Jingmenviruses between multiple tick species could be explained by frequent cofeeding of different tick species on the same mammalian host. This mechanism was described previously as “nonviremic transmission” of arboviruses by Labuda et al. (12). Those authors suggested that infected ticks may transmit a pathogen to uninfected ticks when they aggregately feed onto the same local skin site. This mechanism may be involved in the maintenance of the viral cycle in specific ecological niches.

The ecological characteristics of tick-borne Jingmenviruses led us to set up a serosurvey to assess the prevalence of human and cattle Jingmenviruses in asymptomatic populations highly exposed to infected vectors. No cattle serum positive for JMTV was detected, although previous studies were able to demonstrate the susceptibility of cattle to JMTV infection (2, 9). In contrast, one French human serum sample slightly positive for one ORF of Ixodes ricinus JMTV was detected. Although this result needs to be further confirmed (for example, by implementing a seroneutralization assay) to exclude the possibility of cross-reactions, the phylogenetic proximity of French JMTV to Alongshan virus human pathogen raised the issue of the circulation of Jingmenviruses in French human population. We observed that the seroprevalence of Jingmenvirus infection in asymptomatic populations was very low, as described previously in other studies (3, 6). In addition, the fact that Jingmenviruses were detected only in symptomatic and/or severe human cases (3, 6) suggests that most infections are patent and that asymptomatic infections are rare. However, the wide geographical distribution of Jingmenviruses and their ability to infect numerous vertebrate and invertebrate hosts indicate the need to deeply monitor the circulation of Jingmenviruses, especially if the conditions for virus transmission between ticks and mammals change in a manner that results in better vector capacity.

Analyses of the worldwide geographical distribution of JMTV need to take into account possible JMTV infection in returning travelers presenting with arbovirus-like symptoms. Further studies are also needed to understand the biology and ecology of Jingmenviruses to determine which characteristics, at the virus, tick, and host levels, can explain the development of symptomatic infections by Jingmenviruses.

## MATERIALS AND METHODS

### Sample collection and preparation of metatranscriptomics libraries. (i) Processing of ticks.

A total of 1,450 Ixodes ricinus nymph ticks and 555 I. ricinus adult ticks were collected in France (in the Alsace and Ardennes regions, respectively) as previously published (24), by flagging in areas where contacts with humans and domestic animals are frequently reported. All ticks were washed to remove external contaminants (25), nymphs were pooled into groups of 25 individuals, and all samples were homogenized in Dulbecco’s modified Eagle’s medium (DMEM) supplemented with 10% of fetal calf serum (Invitrogen, Paris, France). After clarification, DNA and RNA were extracted from supernatant using a Macherey-Nagel NucleoSpin tissue kit and a NucleoSpin RNA II kit, respectively, according to the recommendations of the manufacturer (Macherey-Nagel, Hœrdt, France) (24, 26). Pools of total RNA were constituted and used as the template for reverse transcription using random hexamers followed by random amplification using a Qiagen QuantiTect whole-transcriptome kit (Qiagen, Courtaboeuf, France). cDNA was used for library preparations and sequenced on an Illumina HiSeq 2000 sequencer in a single-read 100-bp format outsourced to DNAVision (Charleroi, Belgium) or Integragen (Evry, France).

A total of 312 adult ticks (*n* = 137 Amblyomma variegatum and *n* = 175 Rhipicephalus microplus) were sampled in Guadeloupe and a total of 285 adult ticks (all Rhipicephalus microplus ticks) in Martinique between February 2014 and March 2015. Ticks were processed as previously described (27) (Gondard et al., unpublished) except that total nucleic acid (NA) was extracted from individual ticks by the use of a Macherey-Nagel NucleoSpin 96 virus core kit and a Biomek4000 automatic platform (Beckman Coulters, Villepinte, France). Samples were pooled, and DNA was digested using a Turbo DNA-free kit (Invitrogen) according to the manufacturer’s instructions. Purified RNA was used as the template for reverse transcription performed using random hexamers followed by random amplification using a Qiagen QuantiTect whole-transcriptome kit. cDNA were used for library preparations and sequenced on an Illumina NextSeq 500 sequencer in paired-end 2-by-75-bp format outsourced to DNAVision.

Amblyomma testudinarium ticks (30 larvae and 10 nymphs) collected in Lao PDR were homogenized in 400 μl of 1× phosphate-buffered saline (PBS) containing lysing matrix A beads (MP Biomedicals, Illkirch-Graffenstaden, France). Ticks were ground for 6 min at 25 Hz in a TissueLyser II system (Qiagen). Homogenates were centrifuged at 14,000 × *g* for 3 min at 4°C, and 100 μl of supernatant and TRIzol LS (Invitrogen) were used for total RNA extraction. After extraction, residual DNA was digested with 20 U Turbo DNase (Invitrogen). RNA was purified with a RNeasy minikit (Qiagen), analyzed using a Agilent Bioanalyzer, and used as the template for library preparation using a SMARTer stranded total transcriptome sequencing (RNA-Seq) kit–Pico input mammalian kit (Clontech, TaKaRa Bio, Saint-Germain-en-Laye, France), according to the manufacturer’s instructions. Library sequencing was performed on an Illumina NextSeq sequencer in paired-end 2-by-75-bp format outsourced to DNAVision.

**(ii) Processing of bat samples.** A total of 481 Pteropus lylei bats were sampled during monthly captures performed between May 2015 and July 2016 in Kandal Province, Cambodia. Bats were captured using mist nets; handling and sampling were conducted following FAO guidelines (28) with the authorization and under the supervision of agents of the Forestry Administration of Cambodia, Ministry of Agriculture, Forestry and Fisheries. Oral and rectal swabs were collected, and bats were released back into nature after sample collection. Additionally, 1,590 urine samples were collected from plastic sheets deployed under the roosting trees during the same period. Two pools of urine samples, one pool of oral swabs, and one pool of rectal swabs were constituted and clarified at 10,000 × *g* for 15 min. The supernatant was ultracentrifuged at 100,000 × *g* for 1 h before total NA extraction of the pellet was performed using a QIAamp cador pathogen minikit (Qiagen). After extraction, DNA was digested with 20 U Turbo DNase (Invitrogen), and RNA was purified by the use of an RNeasy minikit (Qiagen), analyzed on a Agilent Bioanalyzer, and used as the template for library preparation using a SMARTer stranded total RNA-Seq kit–Pico input mammalian kit (Clontech). Library sequencing was performed on an Illumina NextSeq sequencer in paired-end 2-by-75-bp format outsourced to DNAVision.

### NGS analyses and genome finishing of JMTV-like viruses.

Raw reads were processed with an in house bioinformatics pipeline, as previously described (29), that comprised quality check and trimming, *de novo* assembly, ORF prediction, and BLASTP-based similarity search against the protein Reference Viral Database (RVDB [30]) followed by the validation of viral taxonomic assignment by BLASTP search against the whole protein NCBI/nr database. Confirmed hits were finally mapped onto the Jingmen tick virus SY84 reference genome (GenBank accession numbers NC_024111 to NC_024114) using CLC Genomics package (Qiagen Bioinformatics).

The complete ORFs of JMTV-like viruses were obtained by conventional PCR and Sanger sequencing after designing specific primers bracketing the missing sequences. Briefly, viral RNA was reverse transcribed using SuperScript IV reverse transcriptase (Invitrogen) and cDNA was subsequently used to amplify lacking portions of the genome of JMTV using Phusion High Fidelity DNA polymerase (New England Biolabs, Evry, France). Positive PCR products were further purified and sequenced by Sanger sequencing (Eurofins Segenic Cochin, Paris, France). When start and stop codons were lacking, rapid amplification of cDNA ends (RACE)-PCR analyses were performed using a 5′/3′ RACE kit (2nd generation) (Roche, Boulogne-Billancourt, France).

### Search for endogenous viral elements (EVE).

In order to identify possible EVEs originating from mammalian or arthropod hosts that might be mistaken for replication-competent viruses, nested qPCR analyses targeting the polymerase (segment 1) gene of JMTV were performed on tick-borne and bat-borne nucleic acids without any RT step. Positive and/or suspicious results were further validated by Sanger sequencing. Accordingly, *in silico* EVE research was performed. Briefly, a homemade database of JMTV-related amino acid sequences was used for a tBlastN search against the genomes of *Ixodes* sp., *Rhipicephalus* sp., *Amblyomma* sp., and *Pteropus* sp. (taxid 6944, 6940, 6942, and 9401, respectively). Positive hits were considered for an E value of <10^−3^.

### Phylogenetic analyses.

Phylogenetic analyses of JMTV-like sequences were constructed with other *Flaviviridae* amino acid sequences retrieved from GenBank targeting the NS5-like gene. Complete and partial open reading frames (ORF) were aligned using MAFFT aligner under the L-INS-I parameter (31). The best amino acid substitution models that fitted the data were determined with ATGC Start Model Selection (32) as implemented in http://www.atgc-montpellier.fr/phyml-sms/ using the corrected Akaike information criterion. Phylogenetic trees were constructed using the maximum likelihood (ML) method implemented through the RAxML program under the CIPRES Science Gateway portal (33) according to the selected substitution model. Nodal support was evaluated using the “automatic bootstrap replicates” parameter.

Nucleotide phylogenetic reconstructions of the four segments were restricted to Jingmenviruses. Complete and partial (>1-kb) nucleotide sequences were retrieved from GenBank (see Table S1 in the supplemental material) and aligned with MAFFT aligner under the L-INS-I parameter or the G-INS-I parameter (31). The best nucleotide substitution models that fitted the data were determined with ATGC Start Model Selection (32) as implemented in http://www.atgc-montpellier.fr/phyml-sms/ using the corrected Akaike information criterion. Bayesian phylogenetic inference (BI) was carried out using MrBayes (34) with two independent runs of four incrementally heated, Metropolis-coupled Markov chain Monte Carlo (MCMC) starting from a random tree (see Fig. S2 in the supplemental material). The MCMC calculations were run for 10 × 10^6^ iterations, and associated model parameters were sampled every 2,000 generations. The initial 20,000 trees in each run were discarded as burn-in samples, and the harmonic means of the likelihood data were calculated by combining the two independent runs.

10.1128/mSphere.00645-19.2 FIG S2 Expanded Bayesian phylogenetic analysis of segment 3. Accession numbers of sequences used in this analysis are provided in Table S1. Posterior probability values above 0.5 are mentioned. Download FIG S2, PDF file, 0.01 MB. Copyright © 2019 Temmam et al. 2019 Temmam et al. This content is distributed under the terms of the Creative Commons Attribution 4.0 International license.

### Serological screening of mammalian sera.

A total of 153 Laotian human blood samples were collected between June 2018 and January 2019 from asymptomatic volunteers (aged 5 years and above) in villages in a remote rural area within Khammouane Province (Lao PDR). Written informed consent was obtained from the participants or their legal guardians. The study protocol was approved by the National Ethics Committee for Health Research (NECHR) in Lao PDR (identifier [ID] 2017.97.NW).

A total of 70 French human serum samples were collected in Alsace from persons with recorded tick bites. Ethics approval was obtained from the Comité de Protection des Personnes d’Ile-de-France VI in 2014, from the National Information Science and Liberties Commission in 2016, and from the French Ministry of Research (DC 2009-1067 collection 25, amendment 2008-68 collection 1).

A total of 178 cattle sera collected in Guadeloupe in 1994 to 1995 and 22 cattle sera collected in 2019 were tested with the LIPS technology. These sera were obtained from other surveillance campaigns approved by the animal owners and the local representative of the French Ministry in charge of agriculture and fisheries.

The LIPS antigens were designed as previously described (29). Extracellular regions of each of the JMTV glycoproteins (GP) were produced as recombinant viral antigens. For French mainland JMTV, both GPs were expressed. LIPS assay was performed as described previously by Burbelo et al. (35, 36) except that human and cattle sera were not diluted. Sera of 30 healthy French volunteers living in the Paris area and not reporting any tick bite (kindly provided by staff members at the ICAReB [Investigation Clinique et Acces aux Ressources Biologiques] Platform of Institut Pasteur, Paris, France) were screened for the presence of antibodies against the targeted viruses as a likely nonexposed group control. Residual background was calculated as the mean of results from 10 negative controls (without serum), and the positivity threshold was defined as the mean of these controls + 5 standard deviations.

### Statistical analyses.

Significant differences between exposed and nonexposed groups of human sera tested by LIPS were calculated using the Student *t* test (IC 95%).

### Data availability.

Complete coding sequences of the four segments of tick-borne and bat-borne Jingmenviruses were deposited into the GenBank database under accession numbers MN095519 to MN095534.
