# Supplementary material for: Insights into the Host Range, Genetic Diversity, and Geographical Distribution of Jingmenviruses
Source: mSphere. 2019 Nov 6;4(6):e00645-19. doi: 10.1128/mSphere.00645-19 (PMC6835211; doi:10.1128/mSphere.00645-19)
Supplement: TABLE S1 [file mSphere.00645-19-st001.docx]

| **Virus strain** | **Host category** | **Host species** | **Country** | **Year** | **Segment 1** | **Segment 2** | **Segment 3** | **Segment 4** |
| --- | --- | --- | --- | --- | --- | --- | --- | --- |
| JMTVDJ1-1 | Mosquito | *Armigeres sp.* | China | 2010 | N/A | N/A | KJ001583 * | N/A |
| JMTVX06L | Tick | *Haemaphysalis campanulata* | China | 2010 | N/A | N/A | KJ001591 * | N/A |
| JMTVX39H | Tick | *Haemaphysalis campanulata* | China | 2010 | N/A | N/A | KJ001597 * | N/A |
| JMTVX55 | Tick | *Haemaphysalis flava* | China | 2010 | N/A | N/A | KJ001600 * | N/A |
| JMTVZY1-7 | Tick | *Haemaphysalis flava* | China | 2010 | N/A | N/A | KJ001601 * | N/A |
| JMTVZY2-4 | Tick | *Haemaphysalis flava* | China | 2010 | N/A | N/A | KJ001602 * | N/A |
| JMTVH115-1 | Tick | *Haemaphysalis longicornis* | China | 2010 | N/A | N/A | KJ001584 * | N/A |
| JMTVH115-4 | Tick | *Haemaphysalis longicornis* | China | 2010 | N/A | N/A | KJ001585 * | N/A |
| JMTVH115-5 | Tick | *Haemaphysalis longicornis* | China | 2010 | N/A | N/A | KJ001586 * | N/A |
| JMTVH118-6 | Tick | *Haemaphysalis longicornis* | China | 2010 | N/A | N/A | KJ001587 * | N/A |
| JMTVX114-1 | Tick | *Haemaphysalis longicornis* | China | 2010 | N/A | N/A | KJ001588 * | N/A |
| JMTVX01 | Tick | *Haemaphysalis longicornis* | China | 2010 | N/A | N/A | KJ001590 * | N/A |
| JMTVX08 | Tick | *Haemaphysalis longicornis* | China | 2010 | N/A | N/A | KJ001592 * | N/A |
| JMTVX23 | Tick | *Haemaphysalis longicornis* | China | 2010 | N/A | N/A | KJ001593 * | N/A |
| JMTVX39L | Tick | *Haemaphysalis longicornis* | China | 2010 | N/A | N/A | KJ001598 * | N/A |
| JMTV36 | Tick | *Haemaphysalis longicornis* | China | 2010 | N/A | N/A | KJ001631 * | N/A |
| JMTVWC3 | Tick | *Ixodes granulatus* | China | 2010 | N/A | N/A | KJ001633 * | N/A |
| JMTVH101 | Tick | *Ixodes sinensis* | China | 2010 | N/A | N/A | KJ001630 * | N/A |
| YS102-1 | Tick | *Rhipicephalus microplus* | China | 2010 | KJ001547 * | KJ001555 * | KJ001563 * | KJ001571 * |
| YJ3-3 | Tick | *Rhipicephalus microplus* | China | 2010 | KJ001548 * | KJ001556 * | KJ001564 * | KJ001572 * |
| 10 | Tick | *Rhipicephalus microplus* | China | 2010 | KJ001549 * | KJ001557 * | KJ001565 * | KJ001573 * |
| 84 | Tick | *Rhipicephalus microplus* | China | 2010 | KJ001550 * | KJ001558 * | KJ001566 * | KJ001574 * |
| 85 | Tick | *Rhipicephalus microplus* | China | 2010 | KJ001551 * | KJ001559 * | KJ001567 * | KJ001575 * |
| 164 | Tick | *Rhipicephalus microplus* | China | 2010 | KJ001552 * | KJ001560 * | KJ001568 * | KJ001576 * |
| 204 | Tick | *Rhipicephalus microplus* | China | 2010 | KJ001553 * | KJ001561 * | KJ001569 * | KJ001577 * |
| X84-3 | Tick | *Rhipicephalus microplus* | China | 2010 | KJ001554 * | KJ001562 * | KJ001570 * | KJ001578 * |
| SY84 | Tick | *Rhipicephalus microplus* | China | 2010 | KJ001579 | KJ001580 | KJ001581 | KJ001582 |
| JMTVX27 | Tick | *Rhipicephalus microplus* | China | 2010 | N/A | N/A | KJ001594 * | N/A |
| JMTVX35 | Tick | *Rhipicephalus microplus* | China | 2010 | N/A | N/A | KJ001595 * | N/A |
| JMTVX37 | Tick | *Rhipicephalus microplus* | China | 2010 | N/A | N/A | KJ001596 * | N/A |
| JMTVx41 | Tick | *Rhipicephalus microplus* | China | 2010 | N/A | N/A | KJ001599 * | N/A |
| JMTV11 | Tick | *Rhipicephalus microplus* | China | 2010 | N/A | N/A | KJ001603 * | N/A |
| JMTV12 | Tick | *Rhipicephalus microplus* | China | 2010 | N/A | N/A | KJ001604 * | N/A |
| JMTV16 | Tick | *Rhipicephalus microplus* | China | 2010 | N/A | N/A | KJ001605 * | N/A |
| JMTV19 | Tick | *Rhipicephalus microplus* | China | 2010 | N/A | N/A | KJ001606 * | N/A |
| JMTV33 | Tick | *Rhipicephalus microplus* | China | 2010 | N/A | N/A | KJ001607 * | N/A |
| JMTV34 | Tick | *Rhipicephalus microplus* | China | 2010 | N/A | N/A | KJ001608 * | N/A |
| JMTV39 | Tick | *Rhipicephalus microplus* | China | 2010 | N/A | N/A | KJ001609 * | N/A |
| JMTV47 | Tick | *Rhipicephalus microplus* | China | 2010 | N/A | N/A | KJ001610 * | N/A |
| JMTV48 | Tick | *Rhipicephalus microplus* | China | 2010 | N/A | N/A | KJ001611 * | N/A |
| JMTV49 | Tick | *Rhipicephalus microplus* | China | 2010 | N/A | N/A | KJ001612 * | N/A |
| JMTV52 | Tick | *Rhipicephalus microplus* | China | 2010 | N/A | N/A | KJ001613 * | N/A |
| JMTV53 | Tick | *Rhipicephalus microplus* | China | 2010 | N/A | N/A | KJ001614 * | N/A |
| JMTV60 | Tick | *Rhipicephalus microplus* | China | 2010 | N/A | N/A | KJ001615 * | N/A |
| JMTV62 | Tick | *Rhipicephalus microplus* | China | 2010 | N/A | N/A | KJ001616 * | N/A |
| JMTV67 | Tick | *Rhipicephalus microplus* | China | 2010 | N/A | N/A | KJ001617 * | N/A |
| JMTV69A | Tick | *Rhipicephalus microplus* | China | 2010 | N/A | N/A | KJ001618 * | N/A |
| JMTV69B | Tick | *Rhipicephalus microplus* | China | 2010 | N/A | N/A | KJ001619 * | N/A |
| JMTV72 | Tick | *Rhipicephalus microplus* | China | 2010 | N/A | N/A | KJ001620 * | N/A |
| JMTV84 | Tick | *Rhipicephalus microplus* | China | 2010 | N/A | N/A | KJ001621 * | N/A |
| JMTV90 | Tick | *Rhipicephalus microplus* | China | 2010 | N/A | N/A | KJ001622 * | N/A |
| JMTV112 | Tick | *Rhipicephalus microplus* | China | 2010 | N/A | N/A | KJ001623 * | N/A |
| JMTV118 | Tick | *Rhipicephalus microplus* | China | 2010 | N/A | N/A | KJ001624 * | N/A |
| JMTV123 | Tick | *Rhipicephalus microplus* | China | 2010 | N/A | N/A | KJ001625 * | N/A |
| JMTV129 | Tick | *Rhipicephalus microplus* | China | 2010 | N/A | N/A | KJ001626 * | N/A |
| JMTV138 | Tick | *Rhipicephalus microplus* | China | 2010 | N/A | N/A | KJ001627 * | N/A |
| JMTV141 | Tick | *Rhipicephalus microplus* | China | 2010 | N/A | N/A | KJ001628 * | N/A |
| JMTV157 | Tick | *Rhipicephalus microplus* | China | 2010 | N/A | N/A | KJ001629 * | N/A |
| JMTV29 | Tick | *Rhipicephalus microplus* | China | 2010 | N/A | N/A | KJ001632 * | N/A |
| JMTV85 | Tick | *Rhipicephalus microplus* | China | 2010 | N/A | N/A | KJ001634 * | N/A |
| JMTVningbo | Tick | *Rhipicephalus sanguineus* | China | 2010 | N/A | N/A | KJ001589 * | N/A |
| Haapasaari-18 | Tick | *Ixodes ricinus* | Finland | 2011 | MN10760 | MN107158 | MN107159 | MN107157 |
| Mogiana_MGTV/E8/11 | Tick | *Rhipicephalus microplus* | Brazil | 2011 | JQ289034 * | N/A | JQ289031 * | N/A |
| Mogiana_MGTV/C1/11 | Tick | *Rhipicephalus microplus* | Brazil | 2011 | JQ289035 * | N/A | JQ289033 * | N/A |
| Mogiana_MGTV/C4/11 | Tick | *Rhipicephalus microplus* | Brazil | 2011 | JQ289036 * | N/A | JQ289026 * | N/A |
| Mogiana_MGTV/E4/11 | Tick | *Rhipicephalus microplus* | Brazil | 2011 | JQ289037 * | N/A | JQ289027 * | N/A |
| Mogiana_MGTV/C2/11 | Tick | *Rhipicephalus microplus* | Brazil | 2011 | JQ289038 * | N/A | JQ289028 * | N/A |
| Mogiana_MGTV/E2/11 | Tick | *Rhipicephalus microplus* | Brazil | 2011 | JQ289039 * | N/A | JQ289029 * | N/A |
| Mogiana_MGTV/C8/11 | Tick | *Rhipicephalus microplus* | Brazil | 2011 | JQ289040 * | N/A | JQ289030 * | N/A |
| Mogiana_MGTV/EL/11 | Tick | *Rhipicephalus microplus* | Brazil | 2011 | JQ289041 * | N/A | JQ289032 * | N/A |
| Mogiana_MGTV/V4/11 | Tick | *Rhipicephalus microplus* | Brazil | 2011 | JX390986 | KY523073 | JX390985 | KY523074 |
| RC27 | Mammal | *Piliocolobus rufomitratus* | Uganda | 2012 | KX377513 | KX377514 | KX377515 * | KX377516 |
| Kosovo 2013-17-266 | Mammal | *Homo sapiens* | Kosovo | 2013 | MH133313 | MH133315 | MH133314 | MH133316 |
| Kosovo 2014-C-K14-1C | Mammal | *Homo sapiens* | Kosovo | 2014 | MH133317 | MH133319 | MH133318 | MH133320 |
| Yanggou_YG | Tick | *Dermacentor nuttalli* | China | 2014 | MH688529 | MH688530 | MH688531 | MH688532 |
| Kosovo 2015-A-K15-1A | Mammal | *Homo sapiens* | Kosovo | 2015 | MH133321 | MH133323 | MH133322 | MH133324 |
| HLJ/2015 | Tick | *Ixodes persulcatus* | China | 2015 | MG880118 * | N/A | MG880119 * | N/A |
| JMTV_100 | Tick | *Rhipicephalus microplus* | Brazil | 2015 | MH155907 | MH155905 | MH155906 | MH155908 |
| Mogiana_282 | Tick | *Rhipicephalus microplus* | Brazil | 2015 | N/A | N/A | MH033852 * | N/A |
| Mogiana_307 | Tick | *Rhipicephalus microplus* | Brazil | 2015 | N/A | N/A | MH033853 * | N/A |
| JMTV_293008 | Mammal | *Bos taurus* | Brazil | 2016 | MH155885 * | N/A | N/A | N/A |
| JMTV_976860 | Mammal | *Bos taurus* | Brazil | 2016 | MH155886 * | N/A | N/A | N/A |
| JMTV_Bov02 | Mammal | *Bos taurus* | Brazil | 2016 | MH155887 * | N/A | N/A | N/A |
| JMTV_Bov42 | Mammal | *Bos taurus* | Brazil | 2016 | MH155888 * | N/A | N/A | N/A |
| JMTV_Bov258 | Mammal | *Bos taurus* | Brazil | 2016 | MH155889 * | N/A | N/A | N/A |
| XJ58 | Rodent | *Microtus obscurus* | China | 2016 | MK174251 | MK174244 | MK174230 | MK174237 |
| XJ61 | Rodent | *Microtus obscurus* | China | 2016 | MK174252 * | MK174245 | MK174231 * | MK174238 * |
| XJ77 | Rodent | *Microtus obscurus* | China | 2016 | MK174253 * | MK174246 | MK174232 | MK174239 * |
| XJ155 | Rodent | *Microtus obscurus* | China | 2016 | MK174254 * | MK174247 | MK174233 | MK174240 * |
| XJ335 | Rodent | *Microtus obscurus* | China | 2016 | MK174255 * | MK174248 | MK174234 * | MK174241 * |
| XJ363 | Rodent | *Microtus obscurus* | China | 2016 | MK174256 * | MK174249 | MK174235 * | MK174242 |
| XJ364 | Rodent | *Microtus obscurus* | China | 2016 | MK174257 * | MK174250 * | MK174236 | MK174243 |
| GXTV108 | Tick | *Amblyomma javanense* | China | 2016 | MG703253 | MG703254 | MG703252 | MG703255 |
| JMTV_1 | Tick | *Rhipicephalus microplus* | Brazil | 2016 | MH155892 | MH155890 | MH155891 | MH155893 |
| JMTV_3 | Tick | *Rhipicephalus microplus* | Brazil | 2016 | MH155896 | MH155894 | MH155895 | MH155897 |
| JTMV_5 | Tick | *Rhipicephalus microplus* | Brazil | 2016 | MH155898 * | N/A | N/A | MH155899 * |
| JTMV_11 | Tick | *Rhipicephalus microplus* | Brazil | 2016 | MH155903 * | N/A | MH155900 * | MH155901 * |
| Mogiana_7347 | Tick | *Rhipicephalus microplus* | Brazil | 2016 | N/A | N/A | MH033854 * | N/A |
| Alongshan_H3 | Mammal | *Homo sapiens* | China | 2017 | MH158415 | MH158416 | MH158417 | MH158418 |
| Alongshan_H9 | Mammal | *Homo sapiens* | China | 2017 | N/A | N/A | MH158419 * | N/A |
| Alongshan_H11 | Mammal | *Homo sapiens* | China | 2017 | N/A | N/A | MH158420 * | N/A |
| Alongshan_H18 | Mammal | *Homo sapiens* | China | 2017 | N/A | N/A | MH158421 * | N/A |
| Alongshan_H26 | Mammal | *Homo sapiens* | China | 2017 | N/A | N/A | MH158422 * | N/A |
| Alongshan_H28 | Mammal | *Homo sapiens* | China | 2017 | N/A | N/A | MH158423 * | N/A |
| Alongshan_H30 | Mammal | *Homo sapiens* | China | 2017 | N/A | N/A | MH158424 * | N/A |
| Alongshan_H111 | Mammal | *Homo sapiens* | China | 2017 | N/A | N/A | MH158425 * | N/A |
| Alongshan_H113 | Mammal | *Homo sapiens* | China | 2017 | N/A | N/A | MH158426 * | N/A |
| Alongshan_H125 | Mammal | *Homo sapiens* | China | 2017 | N/A | N/A | MH158427 * | N/A |
| Alongshan_H136 | Mammal | *Homo sapiens* | China | 2017 | N/A | N/A | MH158428 * | N/A |
| Alongshan_H205 | Mammal | *Homo sapiens* | China | 2017 | N/A | N/A | MH158429 * | N/A |
| Alongshan_H215 | Mammal | *Homo sapiens* | China | 2017 | N/A | N/A | MH158430 * | N/A |
| Alongshan_H217 | Mammal | *Homo sapiens* | China | 2017 | N/A | N/A | MH158431 * | N/A |
| Alongshan_H218 | Mammal | *Homo sapiens* | China | 2017 | N/A | N/A | MH158432 * | N/A |
| Alongshan_H350 | Mammal | *Homo sapiens* | China | 2017 | N/A | N/A | MH158433 * | N/A |
| Alongshan_H351 | Mammal | *Homo sapiens* | China | 2017 | N/A | N/A | MH158434 * | N/A |
| Alongshan_H384 | Mammal | *Homo sapiens* | China | 2017 | N/A | N/A | MH158435 * | N/A |
| Alongshan_H566 | Mammal | *Homo sapiens* | China | 2017 | N/A | N/A | MH158436 * | N/A |
| Alongshan_H766 | Mammal | *Homo sapiens* | China | 2017 | N/A | N/A | MH158437 * | N/A |
|  |  |  |  |  |  |  |  |  |
