# Supplementary figures and images for: Insights into the Host Range, Genetic Diversity, and Geographical Distribution of Jingmenviruses
Source: mSphere. 2019 Nov 6;4(6):e00645-19. doi: 10.1128/mSphere.00645-19 (PMC6835211; doi:10.1128/mSphere.00645-19)

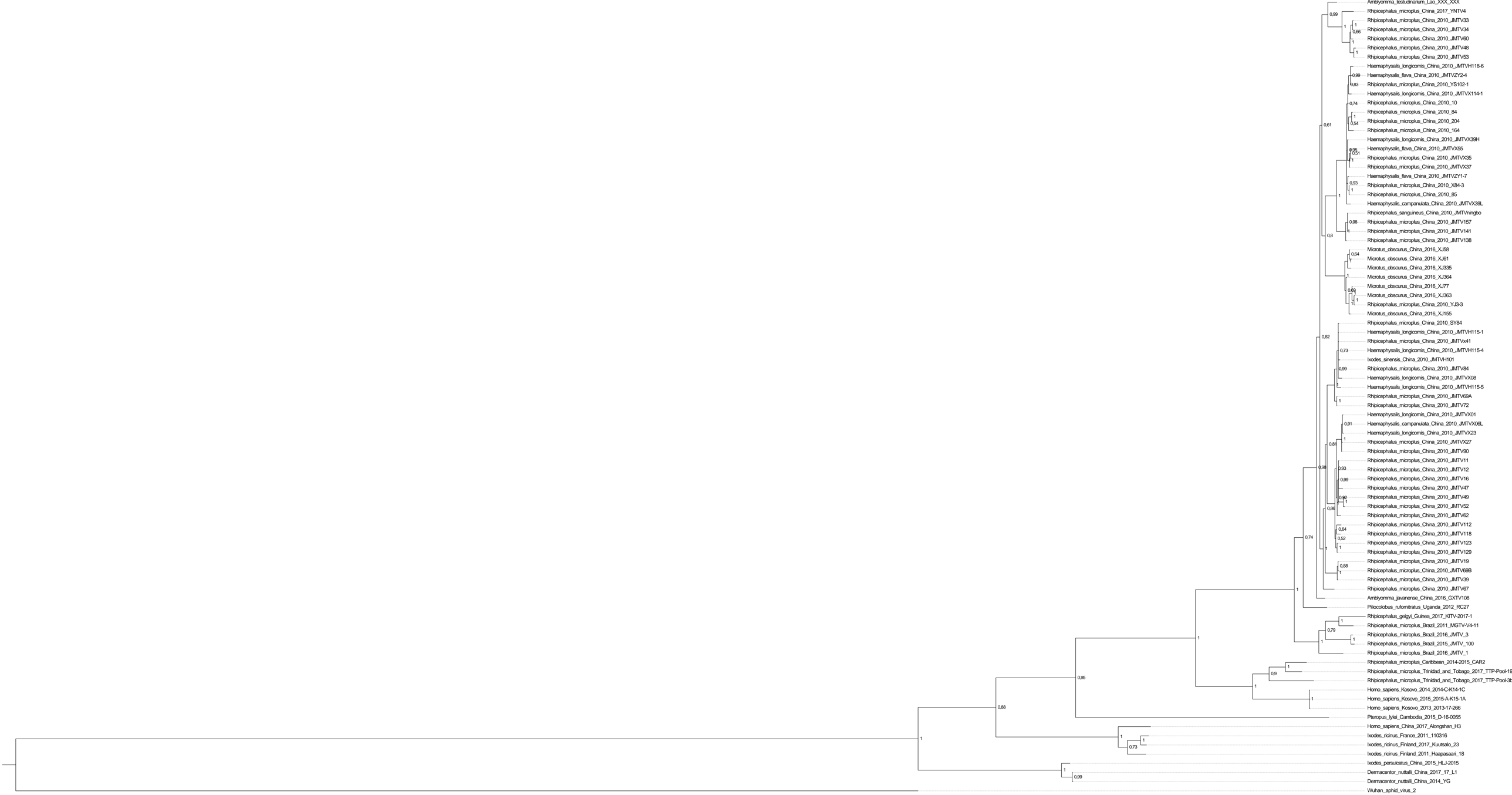

Supplement: FIG S2 [file mSphere.00645-19-sf002.pdf]
